# Recent advances in nanomaterials-based electrochemical sensors for tramadol analysis

**DOI:** 10.5599/admet.1593

**Published:** 2023-04-16

**Authors:** Farideh Mousazadeh, Yar-Mohammad Baghelani, Shamsi Rahimi

**Affiliations:** 1 Department of Chemistry, Payame Noor University, Tehran, Iran; 2 Department of Chemistry, Faculty of Sciences, Ilam University, Ilam, Iran; 3 Department of Chemistry, Payame Noor University, Tehran, Iran

**Keywords:** Tramadol, analytical procedures, nanomaterials, electrochemical sensors

## Abstract

Tramadol is a centrally-acting analgesic used for treating moderate to severe acute and chronic pain. Pain is an unpleasant sensation that occurs most commonly as a result of tissue injury. Tramadol possesses agonist actions at the μ-opioid receptor and effects reuptake at the noradrenergic and serotonergic systems. In the last years, several analytical procedures have been published in the literature for the determination of tramadol from pharmaceutical formulations and biological matrices. Electrochemical methods have attracted tremendous attention for the quantification of this drug owing to their demonstrated potential for quick response, real-time measurements, elevated selectivity and sensitivity. In this review, we highlighted the recent advances and applications of nanomaterials-based electrochemical sensors for the analysis and detection of tramadol, which is extremely important for the indication of effective diagnoses and for quality control analyses in order to protect human health. Also, the main challenges in developing nanomaterials-based electrochemical sensors for the determination of tramadol will be discussed. At last, this review offers prospects for the future research and development needed for modified electrode sensing technology for the detection of tramadol.

## Introduction

Nowadays, the rapid improvements in the medical and pharmaceutical fields increase the diversity and use of drugs. However, problems such as the use of multiple or combined drugs in the treatment of diseases and the insensible use of over-the-counter drugs have caused concerns about the side-effect profiles and therapeutic ranges of drugs and environmental contamination and pollution problems due to pharmaceutical waste. Therefore, the analysis of drugs in various media, such as biological, pharmaceutical, and environmental samples, is an important topic of discussion [1]. Pain is an unpleasant sensation occurring majorly due to tissue damage, influenced by thinking, outlook, behaviour, and community factors. It is responsible for causing emotional and psychological discomfort, too [2]. Long-term use and misuse of opioids causes serious health problems [3,4].

Opioids bind to and activate opioid receptors on cells located in many areas of the brain, spinal cord, and other organs in the body, especially those involved in feelings of pain and pleasure. When opioids attach to these receptors, they block pain signals sent from the brain to the body and reduce the pain sensation. Although a majority of opioids, i.e., morphine, oxycodone, methadone, ketobemidone, tapentadol, tramadol, fentanyl, hydromorphone, sufentanil, buprenorphine and codeine differ in chemical structure, physicochemical properties and in pharmacokinetics, they have one common feature, which is their interaction with the μ opioid receptor as the primary target. Despite this similarity, large differences in the clinical responses (efficacy, effectiveness, toxicity and safety) are seen between the classes of opioids [5]. Opium addicts suffering from opioid withdrawal symptoms are usually managed with tramadol in the initial stage [6].

Tramadol is a 2-(dimethylamino)-methyl-1-(3-methoxyphenyl) cyclohexanol hydrochloride and a 4-phenyl-piperidine analog of opioid drug codeine, which was discovered and synthesized in 1962 by a German company for pain treatment. It was introduced by the name tramadol into the market in 1977 and became available in the US market in 1995 [7]. Opioid-category drugs such as morphine are very potent analgesic but are used less due to their adverse effects of respiratory depression, addiction, dependency, and constipation. The analgesic potency of tramadol is found to be ten times lesser than morphine but is preferred, being safe than the latter. Tramadol is considered safe, as it does not cause respiratory depression and addiction compared to other opioid analgesics [8]. Additionally, tramadol, when administered by the parenteral route, has less abuse potential. Tramadol acts in two different mechanisms. It binds to the μ-opioid receptor, its affinity is weak, and the drug is able to inhibit the reuptake of serotonin and norepinephrine [9]. This drug can be taken by injection, suppository and orally. Most of the reported side effects of tramadol have happened with intramuscular injection and in recent years, its use has increased in society [10]. The tramadol overdose can cause dizziness, vomiting, nausea, diarrhea, chills, hallucinations and respiratory problems since it is considered a toxic material in nature [11]. Therefore, a sensitive method for the measurement of samples containing tramadol is of absolute importance for therapeutic and pharmaceutical reasons.

Analytical chemistry methods refer to techniques used for the detection, identification, characterization and quantification of chemical compounds [12-15]. These methods are commonly used in biology for research, development and quality control of pharmaceutical products. Quantitative determination of tramadol in pharmaceutical and biological samples has been investigated by some instrumental methods such as gas chromatography (GC) [16] gas chromatography-mass spectrometry (GC-MS) [17,18], high-performance liquid chromatography (HPLC) [19], capillary electrophoresis [20,21], spectrophotometry [22,23], chemiluminescence [24] and electrochemical methods [25]. However, most of these methods require highly trained technicians and complex sample preparation that hinders their wide application. Among these methods, researchers considerably focused on electrochemical methods due to the respective benefits such as fast analysis time, higher selectivity, low cost, simplicity, and ability to be miniaturized [26-32].

It is well-known that conventional electrodes have some serious issues due to their sluggish surface kinetics, which severely affects the sensitivity and selectivity of the electrodes. The analytes on the conventional electrodes usually display a broad peak and generally, no peak appears at lower concentrations. The slow electrode reaction of analytes on the bare conventional electrode surfaces requires the high potential to proceed with the reaction at high rates, which greatly exceeds their formal redox potential. The kinetically hindered electrode reactions require a suitable electrocatalyst that can fasten the electrochemical reaction and lower its redox potential. It is obvious that electrode material can play a crucial role in the construction of high-performance electrochemical sensors [33-38]. The researchers are continuously putting their efforts into improving the sensitivity and selectivity of electrochemical sensors.

Nanotechnology refers to the branch of science and engineering devoted to designing, producing, and using structures, devices, and systems by manipulating atoms and molecules at the nanoscale, i.e., having one or more dimensions of the order of 100 nanometres or less. Nanotechnology is used to revolutionize many technology and industry sectors: electronics, energy, material science, medicine, transportation, transportation, and environmental science, to name a few [39-44]. The reasons for their growing applications relate to their unique physical and chemical properties, which are entirely different from their bulk counterparts [45].

Recently more attention has been paid to the synthesis of various nanomaterials to achieve not only ultrasensitive but selective electrochemical sensors [46]. Electrochemical sensors and biosensors integrated with nanostructured materials were employed as powerful analytical devices owing to numerous advantages such as rapid response, high performance, cost-effective, high sensitivity and selectivity by signal amplification not only via the catalytic activity and high conductivity but also by facilitating the immobilization of chemical and biological reagents on the sensor surface [47-49]. The development of nanomaterials has proven fundamental for the development of electrochemical sensors to be used in different application fields such as biomedical, environmental, and food analysis [50-52].

In this review, we mainly focused on the current progress and application of nanomaterial-enabled electrochemical sensors for tramadol detection. Here, we classify the current research based on the types of nanomaterials such as carbon nanomaterials including graphene (Gr) and its derivatives, carbon nanotubes (CNTs), and graphitic carbon nitride), metal nanoparticles (NPs) and metal oxide NPs) used in electrochemical tramadol sensors. Also, the challenges and prospects were discussed in this review.

## Electrochemical sensors based on nanomaterials for detection of tramadol

### Electrochemical sensors based on carbon nanomaterials

Carbon-based nanomaterials exhibit distinct physical and chemical properties besides displaying great potential for applications in material preparation, energy storage, environmental science, pharmaceutical analyses, and medical science [53,54]. Also, carbon nanomaterials as the most widely studied nanomaterials due to outstanding properties, such as unique chemical/physical stability, high heat resistance and corrosion resistance, wide electrochemical windows, large specific surface area, and ultra-high electrical conductivity, playing an extremely important role in the construction of electrochemical sensors for electroanalysis [55]. Electrochemical sensors based on different functions and structures of carbon nanomaterials for electrochemical detection have been established over recent years, including CNTs, Gr and its derivatives, graphitic carbon nitride, etc.

### Electrochemical sensors based on (Gr) and its derivatives

Since the experimental discovery of Gr in 2004 by Novoselov *et al.* [56], it has been extensively employed in various fields [57-59]. Gr is composed of single-layer carbon atoms forming a two-dimensional (2D) crystal structure. The inert electrochemistry, wide potential window, and electrocatalytic properties of this 2D material make it a promising candidate as a sensing element in electrochemical sensors to detect various target analytes. Besides, the high surface area to volume ratio of Gr improves the sensitivity of such electrochemical sensors [60].Afkhami *et al.* [61] reported the preparation of NiFe_2_O_4_/Gr nanocomposite and its application as a modifier for the fabrication of an electrochemical sensor for the simultaneous determination of tramadol and acetaminophen. The decoration of Gr with NiFe_2_O_4_ NPs can provide excellent electrochemical platforms for tramadol and acetaminophen analysis due to the combination of the enlarged active surface area, strong adsorptive capability of the nanomaterial and their specific interactions ability. By using square wave voltammetry (SWV), the peak currents of tramadol and acetaminophen increased linearly with their concentration in the range of 0.01 to 9 μM. The detection limit for their determination was found to be 0.0036 and 0.0030 μM, respectively. The capability of the proposed sensor NiFe_2_O_4_-Gr modified carbon paste electrode (CPE) was investigated by direct analysis of tramadol and acetaminophen in the commercial pharmaceutical samples (acetaminophen tablet, tramadol tablet, and ultracet tablet) and also in the biological fluids (human serum, and urine). Good results for real sample analysis were obtained.

Manokaran *et al.* [62] functionalized nitrogen-doped Gr using poly(diallyldimethyl ammonium chloride) (PDDA-NGr). Then, they anchored Pt-Pd bimetallic NPs on PDDA-NGr to form Pt-Pd/PDDA-NGr nanocomposite. The simultaneous determination of tramadol and acetaminophen was carried out using Pt-Pd/PDDA-NGr modified glassy carbon electrode (GCE). Two well-defined voltammetric peaks were obtained in SWV measurements. The modified electrode detected acetaminophen over a wide linear concentration range from 5.0 to 100.0 μM and tramadol from 12.0 to 240.0 μM. The detection limits were found to be 0.18 and 5.7 μM for acetaminophen and tramadol, respectively. Finally, the amounts of acetaminophen and tramadol present in the human serum sample were analyzed by standard addition method using the Pt-Pd/PDDA-NGr modified GCE.

Mohamed *et al.* [63] constructed a voltammetric sensor based on graphene oxide (GO) and multiwalled carbon nanotubes (MWCNTs) composites (GO-MWCNTs) for the sensitive determination of tramadol [64]. The electroanalytical sensitivity for the determination of tramadol using the GO-MWCNTs/CPE sensor was significantly improved over that of an unmodified CPE. The linear response obtained for tramadol using the GO-MWCNTs/CPE was found to be over the range of 2.0×10^-9^ to 1.1×10^-3^ M with good linearity and high correlation (0.9996). Also, the limits of detection and quantification were found to be 1.50×10^-10^ M and 4.99×10^-10^ M, respectively. In addition, the GO-MWCNTs/CPE sensor enabled the sensing of tramadol in the presence of the frequently co-formulated drug ketorolac tromethamine and acetaminophen without any interference.

Rokhsefid *et al.* [64] prepared a selective and sensitive electrochemical sensor by modifying CPE with Au NPs-Gr nanosheets nanocomposite and 4-hydroxyl-2-(triphenylphosphonio)phenolate (HTP). The modified electrode (Au NPs-Gr-HTP/CPE) showed a successful application for determining tramadol and simultaneous detection of tramadol and acetaminophene. By modification of CPE with Au NPs-Gr-HTP, the oxidation of tramadol was significantly improved. According to differential pulse voltammetry (DPV) measurements, Au NPs-Gr-HTP/CPE showed a wide linear dynamic range (1.0–16.0 μM, and 16.0–100.0 μM) for the determination of tramadol. The detection limit of 0.82 μM for tramadol was obtained. In addition, the analytical performance of the prepared sensor was assessed to detect tramadol and acetaminophen in human urine and pharmaceutical samples with acceptable outputs.

The simultaneous determination of tramadol, codeine and caffeine by using CeO_2_-SnO_2_/rGO nanocomposite-modified GCE was reported by Hosseini *et al.* [65]. The CeO_2_-SnO_2_/rGO/GCE could shift the oxidation potential of tramadol, codeine and caffeine toward a less positivepotential; however, a small amount, but also boosted their oxidation peak current remarkablywhen compared to rGO/GCE, SnO_2_-rGO/GCE, and bare GCE. The simultaneous determination of three analytes was performed on CeO_2_-SnO_2_/rGO/GCE. In optimum conditions, a dynamic range of 0.008-10 μM and 10-270 μM for tramadol, 0.01-12 μM and 12-260 μM for codeine, 0.01-14 μM and 14-260 μM for caffeine with the detection limit of 0.0056, 0.0053, and 0.0055 μM for tramadol, codeine and caffeine, respectively, were obtained. Finally, the presented electrode was successfully applied for the measurement of tramadol, codeine and caffeine in urine and human plasma spiked samples.

Saichanapan *et al.* [66] reported the voltammetric determination of tramadol using a hierarchical graphene oxide nanoplatelets modified GCE (H-GONPs/GCE). The H-GONPs/GCE showed a faster charge transfer rate and larger active surface area. The anodic current response of tramadol was three times higher at the H-GONPs/GCE than at the GONPs/GCE. In the optimal condition, by using adsorptive anodic stripping voltammetric (AdASV), the calibration curve of tramadol demonstrated good linearity in two tramadol concentration ranges (0.05–5.0 μM, and 5.0–20 μM). A higher detection sensitivity (20.7 μA μM^-1^ cm^-2^) and a lower limit of detection (0.015 μM) were obtained. Finally, the developed sensor was applied to detect tramadol in pharmaceutical samples and spiked beverage, saliva, and urine samples.

### Electrochemical sensors based on CNTs

CNTs are nanomaterials formed from one or more Gr sheets rolled cylindrically from their axis, forming tubular structures with a diameter in the nanometer and a length range ranging from micrometers to centimeters [67]. Conceptually, CNTs can be divided into two groups: single-walled carbon nanotubes (SWCNTs) and MWCNTs. SWCNTs are made up of only one sheet of Gr and have a diameter ranging from 1 to 5 nm. However, the synthesis methods currently employed to produce only a small fraction of SWCNT increase its cost and make it difficult to be applied on a large scale. MWCNTs are formed by a set of two or more concentrically coiled Gr sheets, which may have diameters of 10–50 nm [68,69]. Recently, CNTs have been applied in various fields [70-73]. While they have many of the same properties as other types of carbon, CNTs offer unique advantages, including enhanced electronic properties, a large edge plane/basal plane ratio, and rapid electrode kinetics. Therefore, CNT-based sensors generally have higher sensitivities, lower limits of detection, and faster electron transfer kinetics than traditional carbon electrodes [74].

Babaei *et al.* [75] constructed a chemically modified electrode based on MWCNTs-modified GCE. The prepared modified electrode was used for the simultaneous determination of acetaminophen and tramadol. They showed that the application of MWCNTs increases anodic peak currents for both acetaminophen and tramadol on the electrode surface. The results indicated that the use of MWCNTs/GCE allows the simultaneous determination of acetaminophen and tramadol with good sensitivity and selectivity. There was a linear relationship between the oxidation peak current and the concentration of acetaminophen over the range of 0.5 to 210 μM and a linear relationship between the oxidation peak current and the concentration of tramadol over the range of 2 to 300 μM. Also, the MWCNTs/GCE exhibited a low limit of detection for acetaminophen (0.085 μM) and tramadol (0.361 μM). The analytical performance of this sensor has been evaluated for the detection of acetaminophen and tramadol in human serum, human urine and some pharmaceutical preparations with satisfactory results.

S. Branch [76] introduced a voltammetric sensor based on MWCNTs-modified GCE for the determination of tramadol. The MWCNTs/GCE facilitated the determination of tramadol with good sensitivity and selectivity. The DPV experiments of various concentrations of tramadol showed two linear dynamic ranges. The first linear dynamic range was from 4 μM to 35 μM, and the second linear dynamic range was between 60 μM to 550 μM. A detection limit of 0.38 μM was obtained. Finally, the proposed sensor was used in the determination of tramadol in some real samples like a human serum, urine and some drugs, without the necessity of sample pretreatments or time-consuming extraction, with satisfactory results.

Atta *et al.* [77] fabricated the modified GCE by electrodeposition of mono-dispersed Au NPs onto CNTs. The electroanalysis of tramadol was investigated at the surface of the modified electrode by different electrochemical techniques. The increase of the electroactive surface area and the synergistic electrocatalytic activity were achieved by combining AuNPs with CNTs responsible for the modified GCE's improved performance. The modified electrode showed a low detection limit (68 nM) and wide linear concentration range (0.1–1000 μM) for the detection of tramadol concentration. Tramadol was successfully determined in pharmaceutical dosage forms without any pretreatment of the samples.

An amplified sensor based on improved CPE with 1,3-dipropylimidazolium bromide and MgO/SWCNTs nanocomposite (1,3-di-Br/MgO/SWCNTs/CPE) for tradamol determination was prepared by Hosseini *et al.* [78]. The 1,3-DIBr/MgO/SWCNTs/CPE showed good catalytic ability for the determination of tramadol. SWV investigation showed a linear relationship between the tramadol current and concentration within the range of 0.05-280 μM with a detection limit of 8.0 nM. Finally, the modified electrode was successfully used for determination of tramadol in the injection and urine samples.

Foroughi *et al.* [79] developed an electrochemical sensor based on La^3+^ doped fern-like CuO nanoleaves/MWCNTs modified GCE for voltammetric determination of tramadol. Fern-like La^3+^-CuO nanoleaves and MWCNTs significantly improved the sensitivity of the sensor. A limit detection of 0.014 μM within a linear range of 0.5-900.0 μM was determined for obtaining the quantitative tramadol detection. Finally, the developed sensor was utilized to determine tramadol and acetaminophen in pharmaceutical formulations and urine samples, with successful results.

Atta *et al.* [80] modified a CPE with Co_3_O_4_ NPs, ionic liquid (IL) and CNTs in the presence of sodium dodecyl sulfate (SDS). The modified electrode (CNTsILCo_3_O_4_-SDS/CPE) was applied for the determination of nalbuphine and tramadol narcotic analgesic drugs [42]. They evaluated the effect of the type of metal oxide on the catalytic activity of the sensor. Nano-cobalt oxide showed the highest electrocatalytic activity, excellent conductivity, antifouling ability and charge transfer enhancement compared to other studied metal oxides. Under the optimized conditions, simultaneous determination of nalbuphine and tramadol in human urine using the proposed sensor was successfully achieved with sub-nano detection limits of 0.58 nM and 0.62 nM, respectively. Finally, the practical analytical performance of the sensor was studied for the determination of nalbuphine and tramadol in their pharmaceutical samples with satisfied recovery results.

A voltammetric platform based on NPs of antimony oxide (Sb_2_O_3_ NPs) and MWCNTs was reported by Çidem *et al.* [81]. The prepared nanocomposite was used for the modification of a GCE to the electrooxidation of tramadol. High catalytic activity was observed towards the oxidation of tramadol using the proposed voltammetric platform. The peak current exhibited a linear dependence on the concentration of tramadol in a dynamic range of 4×10^-8^–3.0×10^-5^ M with a detection limit of 9.5×10^-9^ M. Finally, the modified electrode was used for the sensitive determination of tramadol in pharmaceuticals.

Tavana *et al.* [82] synthesized Pt-Pd-doped NiO NPs decorated at the surface of SWCNTs (Pt-Pd/NiO NPs/SWCNTs) using a simple chemical precipitation method and characterized by various methods. Moreover, a highly sensitive electroanalytical sensor was fabricated by incorporating synthesized Pt-Pd/NiO NPs/SWCNT nanocomposites into a CPE in the presence of 1-ethyl-3-methylimidazolium methane sulfonate (EMICH_3_SO_3_^-^) as a binder. The Pt-Pd/NiO NPs/SWCNTs/EMICH_3_SO_3_^-^/CPE showed a powerful electrocatalytic activity for the electrooxidation of nalbuphine and tramadol. The Pt-Pd/NiO NPs/SWCNTs/EMICH_3_SO_3_^-^/CPE also showed good catalytic activity for the determination of nalbuphine in the presence of tramadol and the oxidation potential of these drugs separated with ΔE = 460 mV. The Pt-Pd/NiO NPs/SWCNTs/EMICH_3_SO_3_^-^/CPE was used to determine nalbuphine with a detection limit of 0.9 nM and tramadol with a detection limit of 50.0 nM in drug samples.

The development of a new voltammetric sensor based on molecularly imprinted poly(acrylic acid)-MWCNT nanocomposite (MIP-MWCNT) drop coated onto GCE was reported by Ricardo Teixeira Tarley and co-workers [83]. The prepared sensor was applied to tramadol determination in pharmaceutical samples. The voltammetric sensor prepared by suspension of MIP-MWCNT at 1:1 (w/w) ratio shows an improved performance compared to unmodified GCE. The peak currents were proportional to the tramadol concentration in the range of 9.0 to 30.0 μM. Thus, the calculated limit of detection and limit of quantification to the proposed sensor were 1.4 μM and 4.8 μM, respectively.

The modification of a pencil graphite electrode (PGE) with MWCNTs capped Au NPs for electrochemical determination of tramadol was proposed by Kolahi Ahari and co-workers [84]. The combination of MWCNTs and Au NPs improved the electrocatalytic activity of the sensor toward the oxidation of tramadol in solutions. A linear relationship was observed between DPV response of the modified electrode and the concentration of tramadol in the range of 0.012-0.1 and 0.1-3.0 μM. The detection limit of the method was 0.005 μM. In addition, the proposed sensor (MWCNTs-Au NPs/PGE) showed high sensitivity and selectivity for the determination of the concentration of tramadol in tablets and biological fluids.

Li and Wang [85] worked on the development of a stable, sensitive, and selective electrochemical sensor based on CoO NPs and functionalized carbon nanotubes (CoO@f-CNTs) for the detection of tramadol. The CoO@f-CNTs nanocomposite-modified GCE was made using an electrodeposition approach. The electrochemical studies using DPV and amperometry revealed that f-CNTs and CoO NPs had a synergistic electrocatalytic effect in promoting charge transfer in the oxidation of tramadol as a sensitive and selective sensor with a linear range of 1 to 300 μM. The detection limit and sensitivity were calculated to be 6 nM and 0.44971 μA/μM, respectively. The usefulness and precision of CoO@f-CNTs/GCE for determining tramadol in prepared real samples from urine samples of athlete volunteers were explored. The results showed that the ELISA and amperometric analyses had a high level of agreement.

### Electrochemical sensors based on graphitic carbon nitride

Graphitic carbon nitride (g-C_3_N_4_) emphasized the analog skeleton to graphite, found to be promising and fascinating material containing its sturdy C=N covalent bonds instead of C=C in graphite and the layers that are linked by van der Waals forces [86]. The incorporation of heteroatoms such as nitrogen atoms into carbon-based materials can enhance the properties of existing material whereby the nitrogen atoms act as the strong electron donor sites for catalytic conductivity due to the chemical nature of the nitrogen atom [87,88]. Therefore, g-C_3_N_4_ is an excellent material for use in the surface modification of electrodes [89].

For example, Hassannezhad *et al.* [90] reported an electrochemical sensor based on graphitic carbon nitride-Fe_3_O_4_ (g-C_3_N_4_-Fe_3_O_4_) nanocomposite modified CPE for voltammetric determination of tramadol. The developed g-C_3_N_4_-Fe_3_O_4_/CPE sensor demonstrated better sensitivity for the analysis of tramadol compared to that of the unmodified CPE. The modified electrode showed a rise in peak current and a decrease in the overpotential for the oxidation reaction of tramadol. Under optimized conditions, the proposed electrode showed great detection efficiency for tramadol using DPV over the concentration range of 0.2–14.0 μM and 14.0–120.0 μM and a detection limit of 0.1 μM. The g-C_3_N_4_-Fe_3_O_4_/CPE sensor demonstrated suitable potential to be used for the quantification of tramadol in biological samples (serum, plasma and urine).

### Electrochemical sensors based on metal NPs

Noble metal nanoparticles (mainly Au NPs, Ag NPs, Pt NPs, Pd NPs, Ru NPs and their alloys Au-Ag, Au-Pt, Ag-Pt, Pt-Pd, etc.) possess exceeding advantages over other nanomaterials, including stability, conductivity, biocompatibility, low cytotoxicity and size-related electronic, magnetic and optical properties [91,92]. These metal nanoparticles are helpful for decreasing the overpotentials of the electroanalytical reactions while maintaining the reversibility of the redox reactions. Further, the noble nanoparticles can be easily electrodeposited on any kind of working electrode surface to enhance the overall surface properties.

The efficacy of the Nafion/blended cetyltrimethylammonium bromide (CTAB) protected Au NPs-modified GCE for the electrochemical detection of tramadol in wastewater was studied by Amin and co-workers [93]. Compared with unmodified GCE, the Nafion/CTAB-Au/GCE, due to the synergistic effect, exhibited excellent electrocatalytic activity toward the oxidation of tramadol. The square wave anodic stripping voltammetric (SWASV) analysis was employed to quantify the amount of tramadol. The SWASV response of Nafion/CTAB-Au/GCE was linear over the 0.5-1 μg/mL and 2-12 μg/mL ranges with a detection limit of 3×10^-4^ μg/mL. The Nafion/CTAB-Au/GCE sensor showed good accuracy and reliability.

Hassanvand and Jalali [94] electrodeposited Au NPs and l-cysteine on GCE to prepare a modified electrode for the simultaneous determination of tramadol and acetaminophen. The enlarged surface area of the electrode and high electrocatalytic activity brought about by the nanocomposite were useful in the sensitive and selective determination of tramadol and acetaminophen. The linear dynamic ranges of concentration obtained by SWV, were 0.1-10.7 μM and 0.5-63.5 μM, for acetaminophen and tramadol, respectively. The limit of detection was calculated as 0.03 μM for acetaminophen and 0.17 μM for tramadol. The proposed electrode was used successfully in the simultaneous determination of the drugs in spiked human plasma samples.

Hojjati-Najafabadi *et al.* [95] synthesized Au NPs using a biosynthesized strategy by *Mentha aquatic* extract and characterized by Uv-vis spectroscopic method. The synthesized Au NPs were used as a conductive mediator for the modification of the tramadol electrochemical sensor. The modified paste electrode with Au NPs and 1-butyl-3-methylimidazolium tetrachloroborate (BMTCB) showed high catalytic activity for the determination of tramadol in an aqueous solution. With a detection limit of 6.0 nM, the Au NPs/BMTCB/CPE revealed a linear relationship between tramadol oxidation current and concentration in the range of 0.01–400.0 M. The real sample tests confirmed the good ability of Au NPs/BMTCB/CPE for the determination of tramadol.

### Electrochemical sensors based on metal oxide NPs

Recently, there has been a growing interest in studying the application of metal oxide NPs in various fields due to their attractive physical and chemical properties. Metal oxide NPs with different morphologies have been made through versatile methods. The main functions of metal oxide NPs in electroanalysis involve the large surface-to-volume ratio, high surface reaction activity, and high catalytic efficiency [96]. The use of metal oxide NPs was reported to improve the response time, linear range, detection limit, reproducibility and long-term stability of the biosensors [97]. The strong affinity of metal oxide NPs to the surface of the working electrode can be achieved by various techniques, including physical adsorption, electrodeposition, chemical covalent bonding and electropolymerization [98].

Madrakian *et al.* [99] fabricated an electrochemical sensor for rapid and sensitive determination of tramadol based on Ni-Al layered double hydroxide (Ni-Al LDH)-Fe_3_O_4_ NPs modified GCE. The modified electrode showed good performance for tramadol determination. Under the optimized conditions, the anodic peak current was linear for the concentration of tramadol in the range 1.0–200.0 μM with a detection limit of 3.0×10^-1^ M. In addition, the method electrode was successfully used to detect the concentration of tramadol in human serum and urine samples.

Memon *et al.* [100] reported the development of an effective and sensitive modified electrode for the quantitative determination of tramadol. This modified electrode was fabricated by modification of GCE with Co_3_O_4_ NPs. The Co_3_O_4_ NPs over the GCE surface resulted in the production of catalytic current, which helped in the development of a sensitive and effective detection tool. Using the modified GCE, under optimized conditions, the determination of tramadol was obtained with a linear range of 0.5–45 μM and a detection limit of 0.001 μM. The modified electrode was successfully applied for the quantitative analysis of tramadol in commercial pharmaceutical samples.

Arabali *et al.* [101] modified a PGE using CuO NPs/ polypyrrole (ppy) nanocomposite as a powerful electrochemical strategy for the determination of tramadol. The CuO NPs-ppy/PGE showed excellent electrocatalytic activity for oxidation determination of tramadol with improving oxidation current about 4.55 times. Using SWV investigation, the over-potential oxidation of tramadol was decreased by about 40 mV compared to the oxidation potential of a drug at unmodified PGE. In the analytical investigation using the SWV method, the CuO NPs-ppy/PGE showed more advantage for the determination of tramadol in the concentration range 5.0 nM–380 μM with a detection limit of 1.0 nM. In addition, the PGE/CuO-NPs/pPy showed acceptable data for the determination of tramadol in drug samples.

Khairy and Banks [102] demonstrated the applicability of screen-printed electrodes (SPE) modified with Yb_2_O_3_ NPs for individual and simultaneous determinations of acetaminophen and tramadol drugs. The acetaminophen and tramadol exhibited non-overlapping voltammetric signals at voltages of +0.30 and +0.67 V (vs. Ag/AgCl; pH = 9) using Yb_2_O_3_-SPEs. Pharmaceutical dosage forms and spiked human fluids were analyzed in wide linear concentration ranges of 0.25–654 and 0.50– 115 μM with limits of detection of 55 and 87 nM for acetaminophen and tramadol, respectively.

Vazirirad *et al.* [103] constructed a chemically modified CPE based on SnO_2_/α-Fe_2_O_3_ hierarchical nanorods as a simple electrochemical sensor for a sensitive simultaneous determination of dopamine and tramadol. Application of SnO_2_/α-Fe_2_O_3_ hierarchical nanorods as the modifier of the CPE showed high oxidation peak currents for the determination of dopamine and tramadol due to its catalytic effect and high surface area. Using the DPV technique, the sensor showed linear ranges of 0.1-70 μM and 0.5-65 μM and low detection limits of 40 nM and 65 nM toward dopamine and tramadol, respectively. Finally, the analytical performance of the sensor was evaluated for the analysis of dopamine and tramadol in human blood serum and urine with satisfactory results.

Reported electrochemical sensors for the determination of tramadol based on nanomaterials are summarized in Table 1.

## Conclusions

This review gives an overview of the recent advances in the application of nanomaterials in the electrochemical detection of tramadol. Nanomaterials can provide a reliable electrochemical platform for the detection of tramadol. The high surface-to-volume ratio, high conductivity, enhanced electron transfer rate, and simple functionalization process are the main reasons that nanomaterials have gained a lot of attention to construct sensors with high sensitivity. Although nanomaterials-based electrochemical sensors have obvious advantages, they still need to be further studied to increase their sensitivity and selectivity in order to provide powerful and effective tools in pharmaceutical and biological samples.

The mechanism of the electrochemical reaction of tramadol on the modified electrodes and the mechanism of action in which the modified nanomaterials play the role of enhancing the sensing sensitivity and selectivity were not elucidated clearly in most present reports. The specific interaction between the target and the modifier material, and interfaces interactions between the different modifiers are always speculated or suggested due to the direct proof. The changes in the composite of the nanostructures-based modified electrodes are seldom investigated in most research, though these changes may contain important information on reaction mechanisms.

The selectivity of the target analyte of the modified electrodes is an issue that deserves special concern. The selectivity and sensitivity of tramadol sensing can be improved by the use of bio-specific recognition molecules, *e.g.*, antibodies, biological enzymes, nucleic acid aptamers, and etc. However, the performance of such biological molecules is greatly affected by operating conditions, such as temperature, the buffer solution, the pH value of the solution, etc., that will have a fatal effect on the sensitivity, selectivity, and stability of the sensor.

Artificial bio-recognition molecules, such as molecularly imprinted polymers, can overcome the above-mentioned shortcomings of biological recognition molecules. Although conductive monomer molecules are selected as the functional monomers, the conductivity of the resulting polymers synthesized by the functional monomers is not as good as the conductive materials. Combining these with high-conductive materials (novel nanostructures with high surface area and conductivity) is necessary for these molecularly imprinted polymers in order to acquire the desired sensitivity and responsiveness of sensors.

Despite the mentioned limitations and problems, electrochemical sensors could offer a relatively cheap, fast, sensitive, and selective method for online detection or working in complicated matrices. They show great potential for further growth in the near future.

## Figures and Tables

**Table 1.** The summary of the reported electrochemical sensors for determination of tramadol based on nanomaterials.

Electrochemical Sensor	Method	Detection Limit	Linear Range	Ref.
**Carbon nanomaterials**				
Gr and its derivatives				
NiFe_2_O_4_-Gr/CPE	SWV	0.0036 μM	0.01-9 μM	[64]
Pt-Pd/PDDA-NGr/GCE	SWV	5.7 μM	12.0-240.0 μM	[65]
GO-MWCNTs/CPE	DPV	1.50×10^-10^ M	2.0×10^-9^-1.1×10^-3^ M	[66]
Au NPs-Gr-HTP/CPE	DPV	0.82 μM	1.0–16.0 μM and 16.0–100.0 μM	[67]
CeO_2_-SnO_2_/rGO/GCE	DPV	0.0056 μM	0.008-10 μM and 10-270 μM	[68]
H-GONPs/GCE	AdASV	0.015 μM	0.05–5.0 μM, and 5.0–20 μM	[69]
CNTs				
MWCNTs/GCE	DPV	0.361 μM	2-300 μM	[78]
MWCNTs/GCE	DPV	0.38 μM	4-35 μM and 60-550 μM	[79]
Au NPs/CNTs/GCE	DPV	68 nM	0.1–1000 μM	[80]
1,3-DI-Br/MgO/SWCNTs/CPE	SWV	8.0 nM	0.05-280 μM	[81]
La^3+^-CuO/MWCNTs/GCE	DPV	0.014 μM	0.5-900.0 μM	[82]
CNTsILCo_3_O_4_-SDS/CPE	DPV	0.62 nM	-	[83]
Sb_2_O_3_ NPs-MWCNTs/GCE	DPV	9.5×10^-9^ M	4×10^-8^–3.0×10^-5^ M	[84]
Pt-Pd/NiO NPs/SWCNTs/EMICH_3_SO_3_^-^/CPE	SWV	0.9 nM	0.1-750.0 μM	[85]
MIP-MWCNTs/GCE	DPV	1.4 μM	9.0 to 30.0 μM	[86]
MWCNTs-Au NPs/PGE	DPV	0.005 μM	0.012-0.1 and 0.1-3.0 μM	[87]
CoO@f-CNTs/GCE	Amperometry	6 nM	1 to 300 μM	[88]
Graphitic carbon nitride				
g-C_3_N_4_/Fe_3_O_4_/CPE	DPV	0.1 μM	0.2–14.0 μM and 14.0–120.0 μM	[93]
**Metal NPs**				
Nafion/CTAB-Au/GCE	SWASV	3×10^-4^ μg/mL	0.5-1 μg/mL and 2-12 μg/mL	[95]
Au/cysteic acid/GCE	SWV	0.17 μM	0.5-63.5 μM	[96]
Au NPs/BMTCB/CPE	SWV	6.0 nM	0.01–400.0 M	[97]
**Metal oxide NPs**				
Ni-Al LDH-Fe_3_O_4_ NPs/GCE	DPV	3.0×10^-1^ M	1.0–200.0 μM	[101]
Co_3_O_4_ NPs/GCE	DPV	0.001 μM	0.5–45 μM	[102]
CuO NPs-ppy/PGE	SWV	1.0 nM	5.0 nM–380 μM	[103]
Yb_2_O_3_-SPE	DPV	0.087 μM	0.5–5400 μM	[104]
SnO_2_/α-Fe_2_O_3/CPE_	DPV	65 nM	0.5-65 μM	[105]

## Abbreviations

Gr: Graphene
CNTs: Carbon nanotubes
NPs: Nanoparticles
SWV: Square wave voltammetry
CPE: Carbon paste electrode
NGr: Nitrogen doped Gr
PDDA: Poly(diallyldimethyl ammonium chloride)
GCE: Glassy carbon electrode
GO: Graphene oxide
MWCNTs: Multiwalled carbon nanotubes
HTP: 4-hydroxyl-2-(triphenylphosphonio)phenolate
H-GONPs: Hierarchical graphene oxide nanoplatelets
AdASV: Adsorptive anodic stripping voltammetric
SWCNTs: Single-walled carbon nanotubes
1,3-DI-Br: 1,3-Dipropylimidazolium Bromide
SDS: Sodium dodecyl sulfate
EMICH_3_SO_3_^-^: 1-ethyl-3-methylimidazolium methane sulfonate
MIP: Molecularly imprinted polymer
PGE: Pencil graphite electrode
f-CNTs: Functionalized carbon nanotubes
CTAB: Cetyltrimethylammonium bromide
SWASV: Square wave anodic stripping voltammetric
BMTCB: 1-butyl-3-methylimidazolium tetrachloroborate
LDH: Layered double hydroxide
ppy: Polypyrrole
